# Evaluation of four rapid diagnostic tests for canine and human visceral Leishmaniasis in Colombia

**DOI:** 10.1186/s12879-019-4353-0

**Published:** 2019-08-27

**Authors:** Giovanny Herrera, Adriana Castillo, Martha S. Ayala, Carolina Flórez, Omar Cantillo-Barraza, Juan David Ramirez

**Affiliations:** 1 Grupo de Investigaciones Microbiológicas-UR (GIMUR), Departamento de Biología, Facultad de Ciencias Naturales y Matemáticas, Universidad del Rosario, Bogotá, Colombia; 2 Grupo de Parasitología, Instituto Nacional de Salud, Bogotá, Colombia; 3 Grupo Biología y Control de Enfermedades Infecciosas (BCEI), Sede de Investigación Universitaria, Universidad de Antioquia, Medellín, Colombia

**Keywords:** Visceral Leishmaniasis, Immunochromatographic test, Indirect immunofluorescence, Sensitivity, Specificity, PCR

## Abstract

**Background:**

Leishmaniasis caused by different species of *Leishmania* affect 98 countries worldwide. Visceral Leishmaniasis (VL) is the mortal clinical presentation of the disease that causes the dead to more than 90% of the patients who suffer it. The diagnosis of VL is made by the direct observation of the parasite in bone marrow, spleen and/or liver aspirates that requires complex proceedings. Therefore, serum samples are submitted to Indirect Immunofluorescence to identify the presence of anti-*Leishmania* antibodies. Despite the variability in the diagnostic performance of the Immunochromatographic Tests (ICTs), there are many evidences that suggest that ICTs can be used for epidemiological screening. However, in Colombia there are not any evidence about the performance of the ICTs for VL diagnosis*,* both for human and canine serum samples. Therefore, this study evaluated the diagnostic performance of 4 ICTs for VL (2 ICTs in human sera and 2 ICTs in canine sera) in samples from endemic areas of Colombia.

**Methods:**

We selected a total of 156 human serum samples (82 positive and 74 negative for VL) and 126 canine serum samples (71 positive and 54 negative) diagnosed by *in house* Indirect Immunofluorescence (IIF). The samples were submitted to the ICTs following the manufacturers’ instructions. Statistical analysis was performed to evaluate the diagnostic performance of each ICT in comparison with the IIF. PCR for HSP70 gene and sanger sequencing was performed in samples with negative results for both ICTs.

**Results:**

The sensitivity (S) of both ICTs for human samples (Ad-bio Leishmania IgG/IgM Combo Rapid Test and Kalazar Detect™) was 91.5% and specificity (E) were 93.2 and 89.2% respectively, while for the ICTs tested on canine samples (Kalazar Detect™ Rapid Test, Canine and DPP® CVL rapid test) we found S values between 82.9 and 85.7% and E values between 79.6 and 92.6%. We found *L. infantum* by PCR and sequencing in 2 human samples, and *L. braziliensis* and *L. amazonensis* in canine serum samples that were negative by both ICTs.

**Conclusions:**

We conclude that both tests evaluated on human samples have a similar diagnostic performance, while the Kalazar Detect™ Rapid Test, Canine showed a better diagnostic performance than the DPP® CVL rapid test evaluated on canine samples. Also, we suggest that it is necessary to design tests with antigens of the circulating strains to increase its diagnostic utility.

## Background

Leishmaniasis comprises a group of diseases caused by parasites of the genus *Leishmania*, and transmitted to humans and other mammals mainly by insects of the family Psychodidae [1–4]. Visceral leishmaniasis (VL) is a deadly form of the disease. It is estimated that more than 500 million people are at risk of acquiring VL worldwide. Most of them live in remote rural areas and the diagnosis is difficult [2, 3]. In the Americas, the disease is endemic in 12 countries with 55,530 new cases between 2001 and 2016 of which 96% were reported from Brazil [5, 6]. Argentina, Brazil and Paraguay have unique characteristics of disease transmission, such that they exhibit expansive transmission, whereas Colombia and Venezuela exhibit stable transmission [7]. However, in recent years, Colombia has experienced an increased number of cases [5]. Between 2008 and 2016, there were 181 cases of VL in Colombia; however, this number might be higher because of miss-diagnosis and the difficulty associated with correct diagnosis of this disease in Colombia, where most cases captured by health systems and epidemiological surveillance correspond to outbreaks in different endemic foci [1, 5].

Although direct observation of parasites from bone marrow aspirate has been recognized as the gold standard for diagnosis of VL, it is restricted to a few medical centers with trained personnel, due to the difficulty of the procedure and the associated risks for patients [1, 4, 8]. Therefore, indirect immunofluorescence (IIF) is used as a reference technique in various countries, including Brazil, Venezuela, Argentina, Paraguay, and Colombia. This technique can be performed in any laboratory using serum samples that can be easily obtained, and demonstrates very good diagnostic performance (sensitivity: 80–100% and specificity: 90–100%) [9–12]. Despite its good performance, in Colombia, its use remains limited to the national reference laboratory, as it also requires special equipment and trained personnel resulting in diagnostic and treatment delays.

In addition, there has been a relatively recent increase in the number of cases of VL in urban areas, where close interactions between various vectors and reservoirs have facilitated the transmission and emergence of outbreaks [1]. In this context, the fundamental role of the dog (*Canis lupus familiaris*) in the urban transmission process has been emphasized, as it is a known reservoir of the *Leishmania* parasite and has close contacts with humans [13, 14]. However, sampling in dogs is not routinely performed, limiting the availability of information regarding its role in *Leishmania* infections in Colombia.

Immunochromatographic tests (ICTs), based on antigens of the *Leishmania donovani* complex, represent an alternative method that is used worldwide in screening for VL. These are used in endemic areas, as they allow presumptive access to rapid diagnosis and are easy to perform [15–17]. A variety of studies have validated the diagnostic performance of this rapid test method, with sensitivity and specificity values between 90 and 100% [9, 15–22]. Notably, ICTs have been developed for detection of anti-*Leishmania* antibodies using a nitrocellulose matrix with recombinant antigens [23]. The most important antigens used on these tests are rK39 and rK28, which are based on the *L. donovani* kinesin and surface proteins, respectively [19, 24]. ICTs have an important limitation, in that they exhibit differential performance based on the geographic region in which they are used; thus, it is necessary to evaluate the diagnostic performance of each ICT in each country before its initial use [25]. In addition, the presence of species other than *L. donovani* has been demonstrated in dogs with VL in Brazil and Colombia [26]; and then, the application of rapid tests for other species should be evaluated to determine the level of diagnostic performance.

In Colombia, there are no comparative studies to determine the diagnostic performance of ICTs that are commercially available, which can ultimately lead to health risks for the population in which the test is applied. Therefore, the present study aimed to evaluate the diagnostic performance of four ICTs for VL in serum samples that were collected from humans and dogs in endemic areas of Colombia (two tests in humans and two in dogs).

## Methods

### Sample selection

For the present study, we selected 156 human serum samples and 124 canine serum samples that were stored in the biobank of the Parasitology Laboratory of the Instituto Nacional de Salud. These samples had been collected from different regions of Colombia between June 2008 and June 2018 for diagnostic confirmation by IIF as part of the epidemiological surveillance program that is performed to facilitate mandatory notification of the disease in this country. The identity of the patients was protected by using coded samples. Only serum samples that had a positive or negative result by IIF for VL were selected; all samples had sufficient volume to perform all tests (approximately 150 μL). Lipemic sera were excluded. No sample size calculation was performed because of the low prevalence of the disease in Colombia.

### Indirect immunofluorescence test

The samples were evaluated following the protocol described by Herrera et al. [26]. Briefly, an in-house indirect immunofluorescence assay test (IIF) was used to determine the anti-*Leishmania* antibodies titer using the crude promastigotes antigen from *Leishmania infantum* MHOM/COL/CL044B following international standards [27, 28]. Samples (both, human and canine sera) were classified as positive if fluorescence was observed at a serum dilution of 1/32 or higher (in-house IIF) [29]. Because of the cross-reactivity of *Leishmania* parasites with *Trypanosoma cruzi*, an independent IIF assay was conducted using fixed epimastigotes from the *T. cruzi* MHOM/CO/01/DA strain as antigen. Samples were classified as positive if epimastigote cytoplasmatic or membrane fluorescence was observed at a serum dilution of 1/32 or higher (*in house* IIF).

### rK39- Immunochromatographic test

A total of four immunochromatographic tests were used in the present study (Kalazar Detect™ (InBios International Inc., Seattle, WA USA), Ad-bio Leishmania IgG/IgM Combo Rapid Test (CTK Biotech, Inc. San Diego, CA, USA), DPP® CVL rapid test (BioManguinhos, Rio de Janeiro, Brazil), Kalazar Detect™ Rapid Test, Canine (InBios International Inc.). All tests were performed independently, in accordance with the manufacturers’ instructions. At the time of assessment, the operator did not know the previous results obtained by IIF.

### DNA extraction, and *Leishmania* species identification

DNA from Human and canine serum samples that were negative for both evaluated ICTs, but positive by IIF, were extracted using the High Pure PCR Template Preparation Kit™ (Roche, Basel, Switzerland), in accordance with the manufacturer’s protocol. Subsequently, the DNA was used for amplification of a gene fragment from the heat shock protein 70 (HSP70) with the primers and conditions described by Patiño et al. [30]. EXOSAP reagent (Affymetrix, Santa Clara, CA, USA) was used to purify the amplification products and then were sequenced by the dideoxy-terminal method. Finally, the sequences were submitted to similarity analysis, comparing them with the HSP70 *Leishmania* sequences deposited in the GenBank database, using BLASTn.

### Statistical analysis

The statistical software OpenEpi: Open Source Epidemiologic Statistics for Public Health [31] was used to construct 2 × 2 tables to determine sensitivity, specificity, and positive and negative predictive values. Binomial confidence limits were calculated for test sensitivity, specificity, and positive and negative predictive values (PPV and NPV). To construct receiver operating characteristic (ROC) curves, Stata 14 software was used [32]. The confidence level was designated as 95% and differences with *p* < 0.05 were considered to be statistically significant.

## Results

### rK39 ICTs

**Fig. 1:**
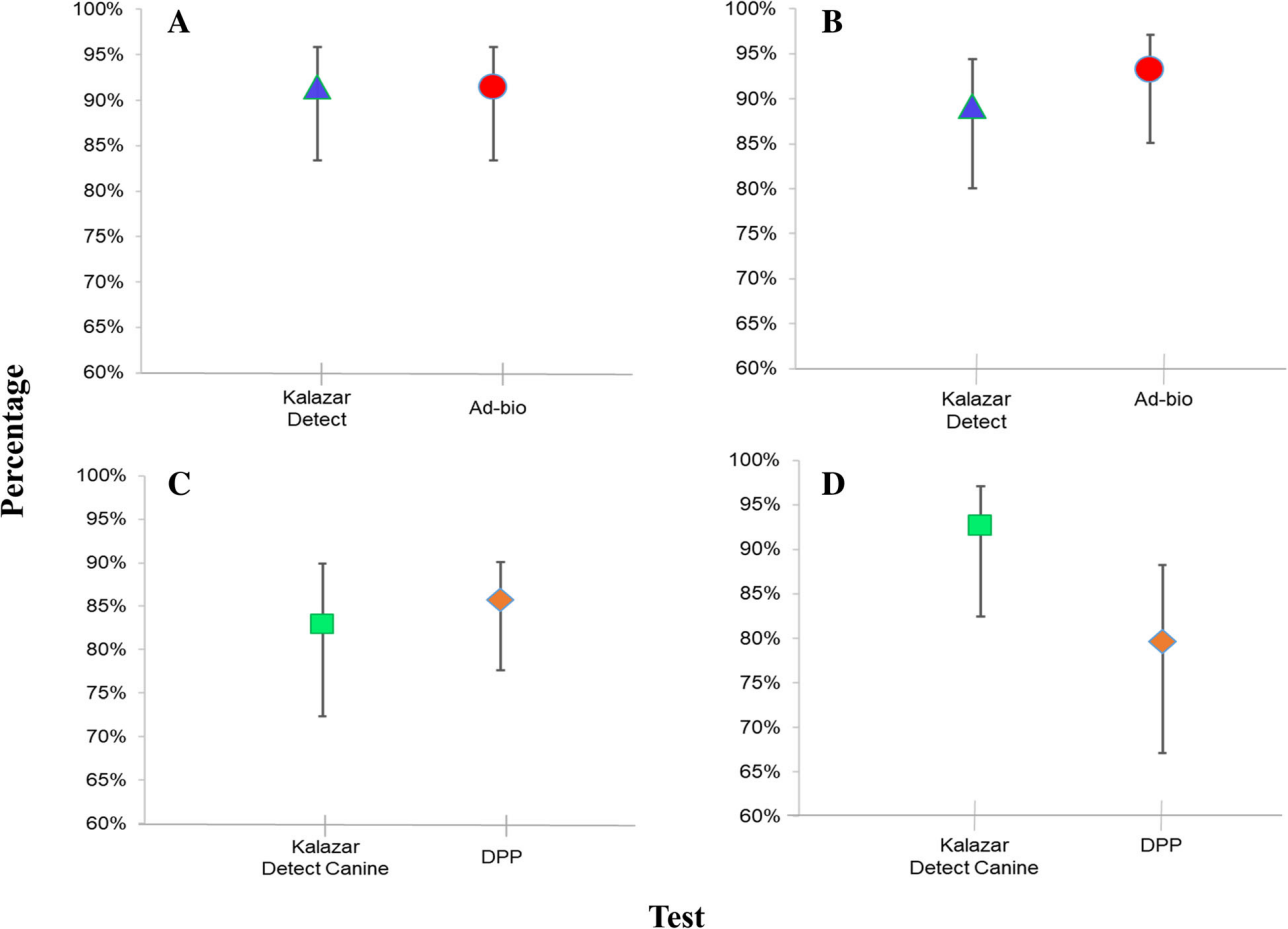
Performance of the visceral leishmaniasis immunochromatographic test (ICTs) evaluated in this study. **a** Sensitivity of ICTs evaluated in human samples. **b** Specificity of ICTs evaluated in human samples. **c** Sensitivity of ICTs evaluated in canine samples. **d** Specificity of ICTs evaluated in canine samples

The tests evaluated in human samples showed a sensitivity of 91.5% (83.4–95.8%). The rapid test Ad-bio Leishmania IgG/IgM Combo Rapid Test showed greater specificity (93.2%), predictive values and likelihood ratios, compared with the Kalazar Detect™ test (Fig. 1a, b; Table 1).

**Table 1:** Performance of rK39 ICTs on Human and Canine samples from endemic areas in Colombia

Parameter evaluated	Human				Canine			
	Kalazar *Detect™*		ad-bio Leishmania IgG/IgM Combo Rapid Test		Kalazar *Detect™* Rapid Test, Canine		DPP® CVL rapid test	
	Value	CI 95%	Value	CI 95%	Value	CI 95%	Value	CI 95%
Sensitivity	91.5%	(83.4–95.8)	91.5%	(83.4–95.8)	82.9%	(72.38–89.91)	85.7%	(75.66–92.05)
Specificity	89.2%	(80.1–94.4)	93.2%	(85.1–97.1)	92.6%	(82.45–97.08)	79.6%	(67.1–88.23)
PPV	90.4%	(82.1–95.0)	93.8%	(86.19–97.3)	93.6%	(84.55–97.46)	84.5%	(74.35–91.12)
NPV	90.4%	(81.5–95.3)	90.8%	(82.2–95.5)	80.6%	(69.15–88.57)	81.1%	(68.64–89.41)
LR (+)	8.46	(6.61–10.84)	13.54	(9.12–20.08)	11.19	(6.80–18.39)	4.21	(3.502–5.056)
LR (−)	0.096	(0.072–0.127)	0.092	(0.069–0.121)	0.185	(0.157–0.219)	0.179	(0.146–0.221)

*PPV* Positive Predictive Value, *NPV* Negative Predictive Value, *LR* Likelihood Ratio

With respect to ICTs evaluated in canine samples, the DPP® CVL rapid test showed a higher sensitivity value (85.7%, compared with 82.9% for the Kalazar Detect™ rapid test, Canine). However, the other parameters were worse, compared with the Kalazar Detect™ rapid test, Canine (Fig. 1c, d; Table 1). There were no associations between the antibody titer detected by IIF and the rapid test result (Table 2). There were not false positive results when IIF for *T. cruzi* was conducted*.*

**Table 2:** rK39 ICTs results by anti-*Leishmania* antibodies titers obtained by IIF

ICT	Result	IIF Result				
		NR	1/32	1/64	1/128	1/256
Kalazar *Detect™*	Positive	8	10	20	22	23
	Negative	66	2	2	2	1
ad-bio Leishmania IgG/IgM Combo Rapid Test	Positive	5	10	20	22	23
	Negative	69	2	2	2	1
Kalazar *Detect™* Rapid Test, Canine	Positive	4	7	7	9	35
	Negative	50	5	0	4	3
DPP® CVL rapid test	Positive	11	7	5	12	36
	Negative	43	5	2	1	2

*NR* No reactive

### ROC curves

**Fig. 2:**
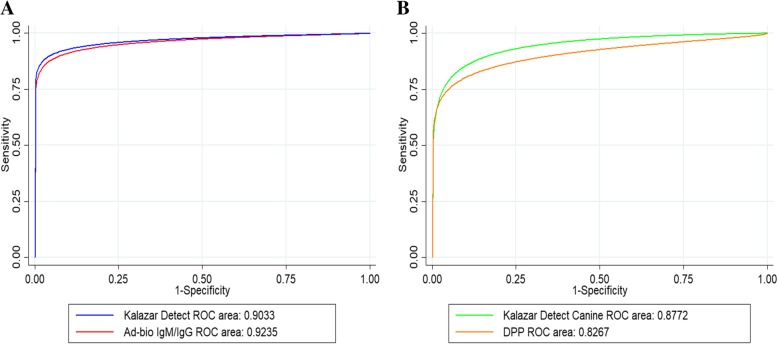
Normalized receiver operating characteristic (ROC) curves of immunochromatographic tests (ICTs) evaluated in this study. **a** Comparison of ROC curves for ICTs evaluated in human samples. **b** Comparison of ROC curves for ICTs evaluated in canine samples

ROC curve analysis revealed no statistically significant differences in the areas under the test curve for humans (*p* = 0.6596) or dogs (*p* = 0.3219) (Fig. 2).

### *Leishmania* species identification

The two serum samples obtained from humans that were negative by both rapid tests demonstrated *L. infantum* infection, while the 3 serum samples from dogs with negative results by both tests demonstrated *L. infantum* [1], *Leishmania amazonensis* [1], and *Leishmania braziliensis* [1] infections.

## Discussion

VL is a global public health problem that causes between 20,000 and 50,000 deaths worldwide [33]. Although 90% of the cases are concentrated within seven countries (Bangladesh, Brazil, Ethiopia, India, Nepal, South Sudan, and Sudan), other countries have experienced multiple cases of the disease, and many are likely undetected by public health surveillance systems [4]. In the Americas, the incidence rate in 2016 was between 1.04 and 4.51 cases per 100,000 people; the poorest people were most affected by the disease [5]. In Colombia, 237 VL cases were reported between 2008 and 2018 in two endemic foci—in the north and center of the country—where departments with optimal conditions for proliferation of vector and parasite, as well as the presence of reservoirs, are well-known [34–38].

In the present study, we sought to determine the diagnostic performance of four rapid tests based on the rK39 antigen for screening of human and canine VL, considering differential performances that these tests have shown according to geographical region [20, 25]. In addition, we considered the recommendations of the WHO for local evaluation of different commercially available tests to determine which showed better diagnostic performance, based on the conditions within each territory [25, 39]. Notably, in Colombia this type of comparison has not yet been performed, although it has been completed in other areas of the world, as well as in the local region (e.g., Brazil and Venezuela) [18, 22, 40–44]. Studies conducted in endemic areas of India and Sudan showed low performance of the tests based on the rK39 antigen, while other antigens (e.g., rKE16) have shown better performance for the detection of anti-*Leishmania* antibodies [45, 46]. Conversely, studies performed in Brazil and Venezuela have shown > 85% sensitivity and specificity in the rapid tests, which supported their use within the screening scheme for VL [40, 42, 44, 47].

With respect to the diagnostic performance in tests of human samples, the sensitivity values found in this study (91.5%) (Fig. 1a) agree with the findings of a Cochrane review by Boelaert et al. [23], who reported a sensitivity value of 91.9%. However, this value differs slightly from the 84.7% reported by the WHO TDR in Brazilian samples using the Kalazar Detect™ test [25]. The presence of false negatives among samples evaluated directly influences the sensitivity and could result in low specific antibody titers; these can occur due to age, nutritional status, and immune status, as well as parasite diversity. [25]. The immune response is an important element to consider when interpreting false negatives, as evasion by the parasite involves different mechanisms that caused reduced antigenic presentation; this modification in the humoral response may generate a reduction in specific antibodies [48]. Likewise, the role of polyparasitism in the development of aberrant immune responses has been described, which may result in low antibody titers [45, 46]. This element could play a fundamental role in patients with VL in Colombia, as many are children living in rural areas where both malaria and Chagas disease, as well as intestinal parasites, have been reported [49].

Intraspecies diversity in *L. infantum* was reported in Colombia by Herrera and collaborators in 2018 [26], who showed that there may be genetic differences among parasites within the *L. infantum* species that are isolated from patients with VL. These genetic differences, along with environmental and nutritional factors that affect the patients, could influence the production of antibodies; this might be mediated by alterations in antigenicity, such as reduction in the immune response [45]. However, more extensive studies are needed with respect to both molecular aspects of the parasite and the general state of health among patients with VL.

With respect to antibody titers, it is particularly important to emphasize that positive samples with different titers classified by IIF were included in the study (Table 2), which when analyzed did not show a differential behavior influenced by this factor. This may be related to the type of antibody that is evaluated by the IIF, as this technique performs overall evaluation of the titers of all anti-*Leishmania* antibodies [29], rather than that of a specific antibody (as is assessed in rapid tests with anti-rK39 antibodies). Importantly, the IIF evaluates total crude antigens of the parasite, in their promastigote form; these do not express the K39 antigen, and therefore antibodies directed against this antigen are not measured by this test [29]. This could lead to the presence of false negatives through rapid tests, even at antibody titers of 1/256 (Table 2).

With respect to the level of specificity, there was differential behavior between the tests: The Ad-bio Leishmania IgG/IgM Combo Rapid Test demonstrated a higher value (Table 1). The specificity values of both tests agreed with those of previous reports, which presented values between 90 and 96% [25]. The amounts of false positives presented by both tests were notable; these could have been due to previous infections that generated antibodies that did not reflect a high titer through the IIF, but were detectable by rapid tests [50, 51]. In this context, uses of both the IIF and rapid tests must be accompanied by a clinical history the clearly supports or does not support diagnosis with VL [51].

In contrast, evaluation of canine samples showed some marked differences between tests with respect to diagnostic performance (Table 1). Sensitivity values of both tests agreed with those reported previously by the manufacturers [44, 47, 52, 53], although the values in the present study tended to be lower. With respect to specificity, both tests showed lower values than previously reported [44, 47, 52, 53]. These parameters may have been influenced by previous asymptomatic infections, as well as the presence of species other than *L. donovani* in these animals. This last aspect has been demonstrated in other geographical regions [54–56], including Brazil and Colombia, where the presence of DNA from *L. amazonensis* and *L. braziliensis* was reported in serum samples from dogs with VL [26, 54, 57]. The presence of the above-mentioned subgenus *Viannia* could lead to negative results in rapid tests for samples that were positive by IIF. Therefore, close monitoring is needed for dogs that live in urban endemic areas, as these dogs have been shown to play fundamental roles in the transmission of the disease, as well as in its expansion from rural to urban areas; this is applicable for VL, as well as other clinical presentations of leishmaniasis [10, 13, 58, 59]. The dog represents a unique challenge in controlling the transmission of the VL, due to its ability to move between rural and urban areas; thus, it is more likely to experience simultaneous infections by different *Leishmania* species, and can serve as a “bridge” that enables presentation of different clinical forms of the disease within endemic populations [35]. Its synanthropic relationship with humans, as well as its uncontrolled reproduction in these areas, produces a reverse dilution effect: an enlarged canine population can increase the parasite population, due to the greater availability of susceptible hosts for their transmission; this can lead to increased transmission to humans [60].

With respect to the ROC curves of the tests evaluated in the present study, the four rapid tests exhibited similar performance; there were no significant differences in the areas under the ROC curves for humans (*p* = 0.6596) or dogs (*p* = 0.3219) (Fig. 2). However, other parameters were assessed in this study: LRs allow global evaluation of the performance of a test without the influence of disease prevalence [61, 62]. With respect to LR (+), the Ad-bio Leishmania IgG/IgM Combo Rapid Test, Kalazar Detect™ Rapid Test, Canine showed values higher than 10 (Table 1) for humans and dogs, respectively, suggesting that these tests are superior for the detection of anti-rK39 antibodies, in comparison with the other tests evaluated. With respect to the LR (−), the tests did not show wide differences (0.096 vs. 0.092 for the tests evaluated in humans; 0.185 vs 0.179 for the tests evaluated in dogs).

Finally, although some tests showed better performance compared with their counterpart (i.e., comparison between human tests or between canine tests), administrative and financial elements must be considered when these tests are implemented. With respect to the Ad-bio Leishmania IgG/IgM Combo Rapid Test, there are difficulties regarding availability in Colombia, since these tests must be imported through a process that may take 3 months; thus, it may be difficult to obtain such tests with reasonable speed in remote regions. However, with respect to cost, this test is less expensive than the Kalazar Detect™ Rapid Test (US $3.80 vs. US $12.00). Therefore, the availability of the tests should be evaluated, along with the delivery times involved in their acquisition. Importantly, the Kalazar Detect™ Rapid Test is both available and provides reliable results. With respect to the tests evaluated in dogs, the DPP® CVL rapid test is not commercially available in Colombia, and its access is restricted to research laboratories; thus, it cannot be used routinely in endemic areas. Based on these limitations with respect to availability, and considering the results presented in the present study, the use of the Kalazar Detect™ Rapid Test, Canine is recommended for use in screening dogs from endemic areas. We note that, although the IT LEISH Individual Rapid Test from BioRad (Hercules, CA, USA) has shown better performance in the Americas [63], it is not available in Colombia. We attempted to import this test; however, due to Colombian regulations, we could not include it within this comparison. Future studies should consider inclusion of the IT LEISH Individual Rapid Test.

## Conclusions

Rapid tests are a valuable tool for the diagnosis of VL, due to their simplicity, low cost, and practical results. Although the evaluated tests showed good performance, no clinical decisions should be made based solely on their results, as it is necessary to evaluate both clinical and epidemiological aspects of patients prior to implementation of therapy. Diagnostic confirmation of all patients, either by microscopy or by IIF, should continue to be mandatory, in order to reduce false positives and negatives. The screening of dogs for VL in endemic areas should be a fundamental facet of the public health surveillance policies of these territories, which can be implemented through the use of rapid tests accompanied by thorough evaluation of the state of the animal before clinical decision-making and public health are considered. Promotion and prevention programs in endemic areas should continue, in order to reduce the number of VL cases, as well as related complications due to late detection. Finally, we encourage governmental authorities to revise the current surveillance guidelines with respect to VL in Colombia due to the limitations encountered in judging whether a patient exhibits VL.

## Data Availability

The data sets used in the current study are available upon request to the corresponding author.

## Abbreviations

ICT: Immunochromatographic tests
IFAT: Indirect Immunofluorescence Assay Test
IIF: Indirect Immunofluorescence
LR: Likelihood ratio
NPV: Negative predictive value
PPV: Positive predictive value
Sp: Specificity
VL: Visceral Leishmaniasis
